# Increased leptin/adiponectin ratio relates to low-normal thyroid function in metabolic syndrome

**DOI:** 10.1186/s12944-016-0403-4

**Published:** 2017-01-11

**Authors:** Lynnda J. N. van Tienhoven-Wind, Robin P. F. Dullaart

**Affiliations:** Department of Endocrinology, University of Groningen and University Medical Center Groningen, Groningen, The Netherlands

**Keywords:** Adiponectin, Leptin, Leptin/adiponectin ratio, Metabolic syndrome, Thyroid function

## Abstract

**Background:**

Low-normal thyroid function within the euthyroid range may contribute to increased atherosclerosis susceptibility. The leptin/adiponectin (L/A) ratio is associated with cardiovascular disease and reflects adipose tissue dysfunction. Relationships of the L/A ratio with low-normal thyroid function are unknown.

**Methods:**

Relationships of thyroid stimulating hormone (TSH) and free thyroxine (free T_4_) with leptin, adiponectin and the L/A ratio in euthyroid subjects were documented in 67 fasting subjects with metabolic syndrome (Mets) and 86 euthyroid subjects without MetS (TSH and free T_4_ levels within the institutional reference range).

**Results:**

Neither plasma leptin nor adiponectin was significantly correlated with TSH or free T_4_ in subjects with and without MetS. In the whole group, high sensitivity C-reactive protein (hs-CRP) was positively correlated with the L/A ratio (*r* = 0.485, *P* < 0.001). Notably, the L/A ratio was positively correlated with TSH in subjects with MetS (*r* = 0.252, *P* = 0.040) but not in subjects without MetS (*r* = −0.068, *P* = 0.54; interaction term, *P* = 0.027). In MetS subjects, the L/A ratio remained positively related with TSH after adjustment for age, sex, diabetes status, hs-CRP and the use of antihypertensive and glucose lowering medication (β = 0.283, *P* = 0.018), as well as after adjustment for individual MetS components (β = 0.294, *P* = 0.020).

**Conclusions:**

In the context of MetS, a higher TSH within the euthyroid range confers an increased L/A ratio, a proposed marker of atherosclerosis susceptibility and adipocyte dysfunction.

## Background

The concept is emerging that low-normal thyroid function, as inferred from a higher thyroid stimulating hormone (TSH) or a lower free thyroxine (free T_4_) within the euthyroid range, may adversely impact several health issues including the development of cardiovascular disorders [1, 2]. In line with this concept, low-normal thyroid function is associated with a greater increased intima media thickness (cIMT), an established biomarker of subclinical atherosclerosis [3, 4]. Furthermore, low-normal thyroid function is associated with increased coronary artery calcification [5] and progression thereof [6], although an association of a high-normal TSH level with increased coronary heart disease risk has been variably reported [7, 8].

Several factors are likely to contribute to the association of (subclinical) atherosclerosis with low-normal function. Low-normal thyroid function relates to higher plasma levels of total cholesterol and atherogenic apolipoprotein B-containing lipoproteins [9], and contributes to enhanced cholesteryl ester transfer from HDL to triglyceride-rich lipoproteins, a pro-atherogenic process [10, 11]. Low-normal thyroid function conveys changes in high density lipoprotein (HDL) anti-oxidative function as well, which conceivably contribute to impaired oxidative stress defense [12]. Interestingly, thyroid function status affects circulating levels of leptin and adiponectin, adipokines which exert pro- and anti-atherogenic properties, respectively [13–15]. Thus, leptin has been reported to decrease and adiponectin to increase after levothyroxine supplementation in subclinical hypothyroidism [16]. These findings provide a rationale to hypothesize that the plasma leptin/adiponectin (L/A) ratio is higher in subjects with low-normal thyroid function. Of note, the L/A ratio may represent a preferential marker compared to leptin and adiponectin alone in predicting incident cardiovascular disease [17, 18]. The L/A ratio is also considered to represent a biomarker of adipocyte dysfunction [19]. Higher plasma leptin and lower adiponectin levels are well known features of the metabolic syndrome (MetS) [20]. As a result, the L/A ratio is elevated in MetS [21–23], which supports the potential clinical relevance to determine relationships of low-normal thyroid function with the L/A ratio in subjects with MetS.

We initiated the present study to determine possible relationships of plasma leptin, adiponectin and the L/A ratio with TSH and free T_4_ in euthyroid subjects with and without MetS.

## Methods

### Subjects

The study protocol was approved by the medical ethics committee of the University Medical Center Groningen, the Netherlands, approved the study. Written informed consent was obtained from the participants, who were aged > 18 years. Type 2 Diabetes Mellitus (T2DM) and non-diabetic subjects were approached by advertisement in local newspapers. T2DM had been diagnosed previously by primary care physicians applying a fasting plasma glucose ≥ 7.0 mmol/l and/or non-fasting plasma glucose ≥11.1 mmol/l as diagnostic criteria. MetS was defined according to NCEP-ATP III criteria [24]. Three or more of the following criteria were required for categorization of subjects with MetS: waist circumference >102 cm for men and >88 cm for women; hypertension (blood pressure ≥ 130/85 mmHg or use of antihypertensive drugs); fasting plasma triglycerides ≥ 1.7 mmol/l; HDL cholesterol < 1.0 mmol/l for men and <1.3 mmol/l for women; fasting glucose ≥ 5.6 mmol/l.

Serum TSH and free T_4_ levels had to be within the institutional reference range, and anti-thyroid peroxidase (anti-TPO) and anti-thyroglobulin (anti-Tg) auto-antibodies had to be absent (see below).

Subjects with a history of cardiovascular disease (CVD), chronic kidney disease (estimated glomerular filtration rate < 60 ml/min/1.73 m^2^ or micro/macroalbuminuria), liver disorders (serum transaminase levels >two times the upper reference limit), as well as current smokers, subjects who used lipid lowering drugs or insulin were also excluded from participation as were pregnant women. The use of anti-hypertensive medication and oral contraceptives was allowed.

Physical examination did not reveal pulmonary or cardiac abnormalities. Body mass index (BMI) was calculated as weight (kg) divided by height (m) squared. Blood pressure was measured after 15 min of rest at the left arm using a sphygmomanometer. The participants were evaluated between 0800 and 1000 h after an overnight fast.

### Laboratory analyses

Serum and EDTA-anticoagulated plasma samples, prepared by centrifugation at 1400 g for 15 min at 4 °C, were stored at −80 °C until analysis. Plasma glucose was measured shortly after blood collection.

Serum TSH (sandwich principle; Roche Diagnostics GmbH., Mannheim, Germany, cat. no. 117314591; reference range 0.5–4.0 mU/l) and free T_4_ (competition principle; Roche Diagnostics GmbH., Mannheim Germany, cat. no. 11731297; reference range 11.0–19.5 pmol/l) were measured with a electrochemiluminescence immunoassay on a Modular Analytics immunoassay analyzer. Anti-TPO and anti-Tg) auto-antibodies were measured with enzyme-linked immunoassays (ImmunoCap cat nos. 14-4508-35 and 14-4507-35, respectively; Phadia, Freiburg, Germany), and were considered to be positive using cut-off values provided by the supplier (anti-TPO antibodies > 60 IU/ml and anti-Tg antibodies > 289 IU/ml).

Plasma leptin and total adiponectin were assayed using commercially available assays (Luminex xMAP technology; Linco Research Inc., St Charles, MO, USA; Lincoplex panel A cat. no. HADK1-61 K-A and panel B cat. no. HADK2-61 K-B) [25]. All intra-assay and inter-assay coefficients of variation were <6 and <8%. High sensitivity C-reactive protein (hs-CRP) was determined was by nephelometry (BNII N; Dade Behring, Marburg Germany) [17].

Plasma total cholesterol and triglycerides were assayed by routine enzymatic methods (Roche/Hitachi cat nos 11875540 and 11876023, respectively; Roche Diagnostics GmbH, Mannheim, Germany). HDL cholesterol was measured with a homogeneous enzymatic colorimetric test (Roche/Hitachi, cat no 04713214; Roche Diagnostics GmbH, Mannheim, Germany). Non-HDL cholesterol was calculated as the difference between total cholesterol and HDL cholesterol. Glucose was measured on an APEC glucose analyzer (APEC Inc., Danvers, MA, USA).

### Statistical analysis

SPSS 22 (version 22.0, SPSS Inc., Chicago, IL, USA) was used for data analysis. Data are expressed as mean ± SD, median (interquartile ranges) or in numbers. Differences between people with and without MetS were determined by unpaired T-tests or Chi-square tests. Because of skewed distribution, triglycerides, hs-CRP, leptin, adiponectin and the L/A ratio were logarithmically transformed to compare between-group differences, and to perform correlation analyses.

Univariate relationships were calculated using Pearson correlation coefficients. Multivariable linear regression analyses were carried out to disclose the independent relationships of the L/A ratio with thyroid function parameters. To determine whether the relationships of thyroid function parameters with the L/A ratio were different between subjects with and without MetS, interaction terms were calculated as the product term between the thyroid function variable of interest and the presence of MetS. Two-sided *P*-values < 0.05 indicated statistical significance.

## Results

We included 67 subjects with MetS and 86 subjects without MetS (Table 1). Forty nine subjects with MetS and 24 subjects without MetS were diagnosed with T2DM (*P* < 0.001). Twenty three subjects with MetS and seven subjects without MetS were taken anti-hypertensive drugs (mostly angiotensin-converting enzyme inhibitors, angiotensin II receptor antagonists and diuretics, alone or in combination) (*P* < 0.001). Metformin and sulfonylurea were used, either alone or in combination, by 28 and 27 subjects with MetS, respectively. Of the subjects without MetS, 10 used metformin and 12 used sulfonylurea. Other glucose lowering drugs were not used. Estrogens were taken by two post-menopausal woman with MetS and by one pre-menopausal woman without MetS.

**Table 1:** Clinical characteristics, glucose, lipids, leptin, adiponectin, the leptin/adionectin (L/A) ratio and thyroid function parameters in 67 subjects with metabolic syndrome (MetS) and in 86 subjects without MetS

	MetS (*n* = 67)	No MetS (*n* = 86)	*P*-value
Age (years)	58 ± 9	56 ± 9	0.10
Sex (men/women)	40/27	52/34	0.92
Systolic blood pressure (mm Hg)	144 ± 18	131 ± 20	<0.001
Diastolic blood pressure (mm Hg)	89 ± 9	81 ± 10	<0.001
BMI (kg/m^2^)	29.8 ± 4.4	25.1 ± 3.3	<0.001
Waist (cm)	105 ± 12	87 ± 11	<0.001
Glucose (mmol/l)	8.6 ± 2.7	6.2 ± 1.4	<0.001
Total cholesterol (mmol/l)	5.54 ± 0.97	5.62 ± 0.97	0.60
Non-HDL cholestrerol (mmol/l)	4.38 ± 0.98	4.07 ± 1.05	0.068
HDL cholesterol (mmol/l)	1.16 ± 0.34	1.55 ± 0.38	<0.001
Triglycerides (mmol/l)	1.96 (1.70-2.52)	1.15 (0.85-1.55)	<0.001
hs-CRP (mg/l)	2.03 (1.26-4.22)	1.02 (0.48-2.25)	<0.001
Leptin (μg/l)	11.5 (5.5-31.9)	4.8 (3.0-11.6)	<0.001
Adiponectin (mg/l)	14.7 (9.2-26.8)	18.2 (14.2-38.8)	<0.001
L/A ratio (μg/mg)	0.83 (0.37-1.85)	0.23 (0.12-0.62)	<0.001
TSH (mU/l)	1.55 ± 0.70	1.66 ± 0.68	0.37
Free T_4_ (pmol/l)	13.9 ± 1.59	13.8 ± 1.47	0.82

Data in mean ± SD or in median (interquartile range). *BMI* body mass index, *hs-CRP* high sensitivity C-reactive protein, *TSH* thyroid stimulating hormone, *free T4* free thyroxine. Logarithmically transformed values of triglycerides, hs-CRP, leptin, adiponectin and the L/A ratio are used for statistical comparisons

Age, sex distribution, TSH and free T_4_ levels were not significantly different between subjects with and without MetS (Table 1). Blood pressure, BMI, waist, plasma glucose and triglycerides were expectedly higher, whereas HDL cholesterol was lower in MetS subjects. Total cholesterol and non-HDL cholesterol were not significantly different between the groups. Plasma hs-CRP was elevated in MetS subjects. Furthermore, leptin was increased and adopinectin was decreased in MetS subjects (Table 1). As a result, the L/A ratio was approximately three-fold higher in MetS subjects.

**Fig. 1:**
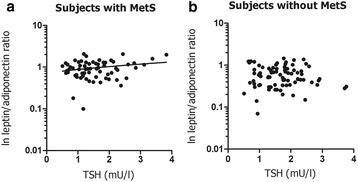
Relationship of the leptin/adionectin (L/A) ratio with thyroid stimulating hormone (TSH in 67 subjects with metabolic syndrome (MetS) (**a**: *r* = 0.252, *P* = 0.040) and 86 subjects without MetS (**b**: *r* = −0.068, *P* = 0.54). The L/A ratio is logarithmically transformed

In the whole group, hs-CRP was correlated positively with leptin (*r* = 0.435, *P* < 0.001) and inversely with adiponectin in univariate analysis (*r* = −0.289, *P* < 0.001). Consequently, hsCRP was correlated positively with the L/A ratio (*r* = 0.485, *P* < 0.001). In the whole group, neither plasma leptin nor adiponectin or the L/A ratio were significantly correlated with TSH or with and free T_4_ (*P* > 0.40 for all; data not shown). Furthermore, neither plasma leptin nor adiponectin were significantly correlated with TSH and free T_4_ in subjects with or without MetS (Table 2). However, the L/A ratio was positively correlated with TSH in MetS subjects, contrasting the lack of such a relationship in subjects without MetS (Table 3; Fig. 1). Indeed, the relationship of the L/A ratio with TSH was different between subjects with and without MetS (interaction term for between group difference: β = 0.201, *P* = 0.027). The positive relation of the L/A ratio with TSH was also present in MetS subjects who did not use antihypertensive drugs (*n* = 43; *r* = 0.329, *P* = 0.034) or metformin (*n* = 39; *r* = 0.285, *P* = 0.070), again without such relationships being found in subjects without MetS (*n* = 79; *r* = −0.026, *P* = 0.82 and *n* = 76, *r* = −0.047, *P* = 0.69, respectively).

**Table 2:** Univariate relationships of leptin, adiponectin and the leptin/adiponectin (L/A) ratio with thyroid stimulating hormone (TSH) and free thyroxine (free T_4_) in 67 subjects with metabolic syndrome (MetS) and in 86 subjects without MetS

	MetS (*n* = 67)		No MetS (*n* = 86)	
	TSH	free T_4_	TSH	free T_4_
Leptin	0.168	0.020	−0.013	−0.113
Adiponectin	−0.090	−0.046	0.154	0.056
L/A ratio	0.252*	0.031	−0.068	−0.106

Pearson correlation coefficients are shown. Logarithmically transformed values of leptin, adiponectin, the L/A ratio are used for statistical comparisons. *P = 0.04

**Table 3:** Multivariable linear regression analysis demonstrating the relationship of the leptin/adiponectin (L/A) ratio with thyroid stimulating hormone (TSH), age, sex and diabetes status and high sensitivity C-reactive protein (hs-CRP) in 67 subjects with metabolic syndrome (MetS; A) and in 86 subjects without MetS (B)

	L/A ratio	
	β	*P*-value
A MetS (*n* = 67)		
Age	−0.130	0.25
Sex (women vs. men)	0.278	0.018
T2DM	−0.049	0.74
hs-CRP	0.150	0.21
TSH	0.289	0.018
B No MetS (*n* = 86)		
Age	−0.076	0.39
Sex (women vs. men)	0.431	<0.001
T2DM	0.002	0.98
hs-CRP	0.430	<0.001
TSH	−0.119	0.22

T2DM: type 2 diabetes mellitus; β: standardized regression coefficient. The L/A ratio and hs-CRP are logarithmically transformed. Models are additionally adjusted for the use of antihypertensive medication, sulfonylurea and metformin

Multivariable linear regression analysis demonstrated that the positive relationship of the L/A ratio with TSH in MetS subjects remained present after adjustment for age, sex, diabetes status and the use of antihypertensive and glucose lowering medication (Table 3A). No such relationship was found in subjects without MetS (Table 3B). In alternative analysis, the L/A ratio was also positively related to TSH in MetS subjects when taking the individual MetS components into account and thus being associated with TSH independent of an enlarged waist (Table 4A). Additionally, the L/A ratio was positively related to an enlarged waist in subjects without MetS (Table 4A and 4B). In the whole group, the L/A ratio was independently associated with female sex (β = 0.338, *P* < 0.001), hs-CRP (β = 0.262, *P* < 0.001) and an enlarged with waist (β = 0.204, *P* = 0.005) without an independent association with TSH (β = 0.079, *P* = 0.214; data not shown).

**Table 4:** Multivariable linear regression analysis demonstrating the relationship of the leptin/adiponectin (L/A) ratio with thyroid stimulating hormone (TSH), age, sex, metabolic syndrome (MetS) components and high sensitivity C-reactive protein (hs-CRP) in 67 subjects with MetS (A) and in 86 subjects without MetS (B)

	L/A ratio	*P*-value
	β	
A MetS (*n* = 67)		
Age	−0.154	0.19
Sex (women vs. men)	0.214	0.082
Elevated glucose	0.053	0.69
Hypertension	0.138	0.25
Enlarged waist	0.201	0.12
Elevated triglycerides	−0.064	0.61
Low HDL cholesterol	0.172	0.17
hs-CRP	0.090	0.47
TSH	0.294	0.020
B No MetS (*n* = 86)		
Age	−0.028	0.76
Sex (women vs. men)	0.53	<0.001
Elevated glucose	0.097	0.31
Hypertension	0.090	0.38
Enlarged waist	0.310	<0.001
Elevated triglycerides	0.177	0.047
Low HDL cholesterol	0.072	0.38
hs-CRP	0.299	<0.001
TSH	−0.060	0.48

*HDL* high density lipoproteins, *β* standardized regression coefficient. The L/A ratio and hs-CRP are logarithmically transformed. Models are additionally adjusted for the use of sulfonylurea and metformin

## Discussion

This study reveals to our knowledge for the first time that the plasma L/A ratio is positively related to a higher TSH level in euthyroid subjects with MetS but not in subjects without MetS. This relationship in MetS subjects remained present when taking relevant covariates into account, including the presence of diabetes, hs-CRP and the use of antihypertensive and oral glucose lowering drugs, and was also present in analysis in which we adjusted for individual MetS components, including an enlarged waist circumference. The current results, therefore, suggest that the L/A ratio, an alleged predictor of cardiovascular disease and biomarker of adipocyte dysfunction [17, 19] associates with low-normal thyroid function. Altogether, the present findings add to accumulating evidence which underscores the possibility that low-normal thyroid function may confer increased atherosclerosis susceptibility [1, 2, 9].

We enrolled strictly euthyroid subjects, as inferred from TSH and free T_4_ levels within the institutional reference range. With this selection criterion, TSH and free T_4_ were similar in subjects with MetS compared to subjects without MetS. This entirely agrees with our previous findings in a small group of non-diabetic subjects [26], although mild thyroid function changes in MetS have been documented in another study [9]. Leptin and adiponectin play an important role in obesity-associated metabolic risk by modulating inflammatory processes and affecting insulin sensitivity [19, 20, 27]. In agreement, we found that the L/A ratio was positively related to hs-CRP in univariate analysis. Given the associations of plasma leptin and adiponectin with (central) obesity, the strong elevations in the L/A ratio in MetS subjects as demonstrated here is not surprising [21–23]. Accordingly, waist circumference predicted the L/A ratio in the present study, even independent of hyperglycemia and other MetS components. We consider this finding reassuring because a considerable number of participants had been diagnosed with type 2 diabetes, making that the number subjects without diabetes was too low to allow for meaningful subgroup analysis.

Clinical observations showing that plasma leptin decreases whereas adiponectin increases after levothyroxine substitution in subjects with subclinical hypothyroidism [16] prompted us to delineate the relationship of the L/A ratio with low-normal thyroid function. In concert with these human findings [16], thyroid hormone upregulates adiponectin gene expression in rat adipose tissue [28]. In contrast, leptin gene expression in rat epididymal fat is downregulated after experimental hyperthyroidism, although lower circulating leptin levels in response to high thyroid hormone exposure are at least in part attributable to a decrease in body fat [29]. It remains to be more precisely determined why there was only a relation of the L/A ratio with TSH in the MetS subjects. In comparison, the relationship with low-normal thyroid function with other pro-atherogenic biomarkers have been demonstrated previously to be particularly evident in diabetic or MetS subjects [2, 9, 10]. Our present observation that this relationship remained present after adjustment for waist circumference would be consistent with a contribution of thyroid function status on this ratio.

A number of other limitations and methodological aspects of our study need to be discussed. First, we performed a cross-sectional study, making that cause-effect relationships cannot be established with certainty. However, we are not aware of published data indicating that the leptin or adiponectin directly affect thyroid hormone regulation. Second, we relied on a single set of thyroid function parameters. In this regard it is noteworthy that each individual probably has a narrow set-point of thyroid function status, underscoring the pathophysiological relevance of once measured thyroid function status [30]. Third, circulating adiponectin increases in response to angiotensin II, making that an effect of the antihypertensive medication used cannot by excluded [31]. Fourth, metformin lowers of the TSH level in hypothyroid subjects and thus could alter the set-point of the pituitary-thyroid axis [32, 33]. Notably, metformin treatment does not elicit TSH changes in euthyroid subjects [33, 34]. For these reasons, we adjusted for the use of antihypertensive and glucose lowering drugs in multivariable regression analysis, confirming the independent relation of the L/A ratio with TSH in MetS. In addition, the positive relation of the L/A ratio with TSH in MetS was also present after exclusion of subjects taking antihypertensive medication or metformin, indicating that the use of these medications did not confound the interpretation of our data.

The interest for a neutraceutical approach to improve the cardiometabolic risk profile besides well-established pharmacological treatment modalities is growing [35]. In this respect it is noteworthy that a combination of several compounds including red yeast rice extract, berberine, policosanol, astaxanthin, coenzyme Q10 and folic acid has been shown to reduce the L/A ratio besides low density lipoprotein (LDL) cholesterol lowering [36]. Given the elevated L/A ratio in MetS, additional studies with respect to a neutraceutical approach to ameliorate pro-atherogenic biomarkers appear to be warranted.

## Conclusions

A higher TSH level within the euthyroid range confers an increased L/A ratio in MetS subjects, which is likely to contribute to an adverse cardiometabolic profile in this patient category.

## Abbreviations

anti-Tg: Anti-thyroglobulin
anti-TPO: Anti-thyroid peroxidase
BMI: Body mass index
cIMT: Intima media thickness
CVD: Cardiovascular disease
free T_4_: Free thyroxine
HDL: High density lipoprotein
hs-CRP: High sensitivity C-reactive protein
L/A ratio: Leptin/adiponectin
LDL: Low density lipoprotein
MetS: Metabolic syndrome
T2DM: Type 2 diabetes mellitus
TSH: Thyroid stimulating hormone
